# Diagnostic accuracy of midkine for hepatocellular carcinoma: A meta‐analysis

**DOI:** 10.1002/mgg3.1071

**Published:** 2019-11-27

**Authors:** Yu Zhang, Juan Tang, Xiao Zhou, Shao‐Liang Zhu, Le‐Qun Li

**Affiliations:** ^1^ Department of Hepatobiliary Surgery Affiliated Tumor Hospital of Guangxi Medical University Nanning China; ^2^ Department of Breast Surgery Affiliated Tumor Hospital of Guangxi Medical University Nanning China

**Keywords:** diagnosis accuracy, hepatocellular carcinoma, meta‐analysis, midkine

## Abstract

**Background:**

There have been many reports on midkine as a promising marker in the diagnosis of hepatocellular carcinoma (HCC). However, the results are inconsistent and even conflicting.

**Methods:**

This meta‐analysis was performed to investigate the accuracy of midkine in the diagnosis of HCC. Meta‐DiSc 1.4 software was used to extract data and to calculate the overall sensitivity, specificity, positive likelihood ratio (PLR), negative likelihood ratio (NLR), and diagnostic odds ratio (DOR). Data are presented as forest plots and summary receiver operating characteristic (SROC) curve analysis was used to summarize the overall test performance.

**Results:**

Ten studies with a total of 753 HCC patients and 977 non‐HCC patients were included. The overall pooled diagnostic data were as follows: the pooled sensitivity of 0.86 (95% confidence interval [CI]: 083–0.88), the pooled specificity of 0.75 (95% CI: 0.73–0.78), the pooled PLR of 4.71 (95% CI: 2.80–7.90), the pooled NLR of 0.18 (95% CI: 0.11–0.30), and the pooled DOR of 36.83 (95% CI: 13.56–100.05). The area under curve value was 0.9266 in the overall SROC curve.

**Conclusion:**

Midkine has moderate diagnostic accuracy for HCC. Due to the design limitations, results inpublished studies should be carefully interpreted. In addition, more well‐designed studies with large sample sizes should be performed to rigorously evaluate the diagnostic accuracy of the MDK.

## Graphical abstract

This meta‐analysis was performed to investigate the accuracy of midkine in the diagnosis of hepatocellular carcinoma.

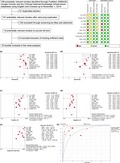

## 1 INTRODUCTION

Hepatocellular carcinoma (HCC) is one of the most common causes of cancer‐related mortality worldwide (Siegel, Miller, & Jemal, 2015). Clinical symptoms of HCC do not show in the early stages but become evident in the late stages, resulting in unsatisfactory curative results. Thus, in order to improve overall survival and prognosis of HCC patients, early detection of HCC for making curative treatments in the early stages is of vital importance (Trinchet et al., 2009). Alpha fetoprotein (AFP) is the commonly used serological marker to detect HCC, however, it is not satisfactory due to the low sensitivity and specificity in the early stages of HCC (Villanueva, Minguez, Forner, Reig, & Llovet, 2010). This highlights the need for new more reliable noninvasive recent biomarkers with better sensitivity and specificity for early detection of HCC.

Midkine (MDK), also known as neurite growth‐promoting factor 2, is a basic heparin‐binding growth factor of low molecular weight. In humans, it is encoded by the MDK gene on chromosome 11 (Ibusuki et al., 2009). It is a developmentally important retinoic acid‐responsive gene product strongly induced during mid‐gestation, hence the name MDK. Expression of the MDK gene in human adult tissues is extremely low and restricted. Mounting evidence has indicated that MDK plays a significant role in carcinogenesis‐related activities, such as proliferation, migration, antiapoptosis, mitogenesis, transformation, and angiogenesis, in many types of solid tumors, including HCCs (Kato, Shinozawa, Kato, Awaya, & Terada, 2000; Muramatsu, 2002). Recently there have been studies reporting the use of MDK as a serum marker for HCC, but the results are heterogeneous and even conflicting. The objective of the present review was to synthesize and analyze the results from systematic selection of research papers that evaluated the diagnostic accuracy of MDK. To the best of our knowledge, this is the first meta‐analysis concerning the accuracy of midkine in the diagnosis of HCC.

## 2 MATERIALS AND METHODS

### 2.1 Search strategy

A comprehensive electronic search in PubMed, EMBASE, Google Scholar, and the Chinese National Knowledge Infrastructure databases using English and Chinese was performed by three independent investigators (YZ, JT, and XZ) for related articles. The latest search was updated on November 1, 2019. The search terms used were as follows: (1) MDK: MDK and midkine; and (2) HCC: HCC, hepatocellular carcinoma, liver cancer, liver cell carcinoma, and hepatic cell carcinoma.

### 2.2 Selection criteria of the studies

The criteria for inclusion were as follows: (a) the studies that investigated the diagnostic accuracy of serum midkine for HCC diagnosis; (b) sample size of HCC and non‐HCC patients, true positive, false positive, false negative, and true negative were reported or calculable. In addition, the criteria for exclusion were used: (a) studies conducted on animals; (b) non‐original papers, such as conference abstracts, letters, and reviews; (c) manuscripts in languages other than English and Chinese (d) duplicate studies; and (e) studies without qualified data or with 20 patients or less.

### 2.3 Data extraction

Data were independently extracted by three authors (YZ, JT, and XZ) from the included studies. The following data were obtained: first author, publication year, country, ethnicity, test methodology, sample size, number of true‐positive, false‐positive, false‐negative, and true‐negative results, cut‐off value, sensitivity, and specificity from the included studies.

### 2.4 Assessment of methodological quality

Quality assessments of included studies were performed by three independent investigators (YZ, JT, and XZ) with Quality Assessment of Studies of Diagnostic Accuracy II (QUADAS‐2) included in systematic reviews checklist recommended by the Cochrane Collaboration Whiting et al. (2011). Each of the signaling questions included to assist in judgments about risk of bias were labeled as ‘‘yes,” ‘‘no,’’ or ‘‘unclear.’’ Each of the items that assessed risk of bias and concerns regarding applicability was labeled as ‘‘high,’’ ‘‘low,’’ or ‘‘unclear.’’ For studies that set healthy controls, if the data of healthy controls could not be removed from the final analysis, the study was regarded as a case‐control design, and the risk bias of the patient selection domain was labeled as ‘‘high risk.’’ Any disagreements in quality assessment were resolved by the third independent investigator (LQL).

### 2.5 Statistical analysis

The recommended standard methods for meta‐analyses of diagnostic test evaluations were used (Devillé et al., 2002). Analyses were performed using Meta‐Disc version 1.4 software programs (Zamora, Abraira, Muriel, Khan, & Coomarasamy, 2006). We pooled sensitivity, specificity, positive likelihood ratio (PLR), negative likelihood ratio (NLR), diagnostic odds ratio (DOR), and their corresponding 95% confidence intervals (CIs) by a random‐effects model. Forest plots were used to depict the overall diagnostic sensitivity and specificity, as well as heterogeneity of eligible studies. Summary receiver operating characteristic (SROC) curves (Moses, Shapiro, & Littenberg, 1993; Walter, 2002) were used to depict the overall diagnostic accuracy of MDK. The extent of heterogeneity was explored using inconsistency index (I‐squared) (Higgins, Thompson, Deeks, & Altman, 2003; Zamora et al., 2006). Additionally, the Spearman correlation coefficient was calculated to verify whether the heterogeneity could be explained by a threshold effect Devillé et al., 2002; Zamora et al., 2006). Meta‐regression was also performed to explain the source of the observed heterogeneity.

## 3 RESULTS

### 3.1 Characteristics of included studies

**Figure 1:**
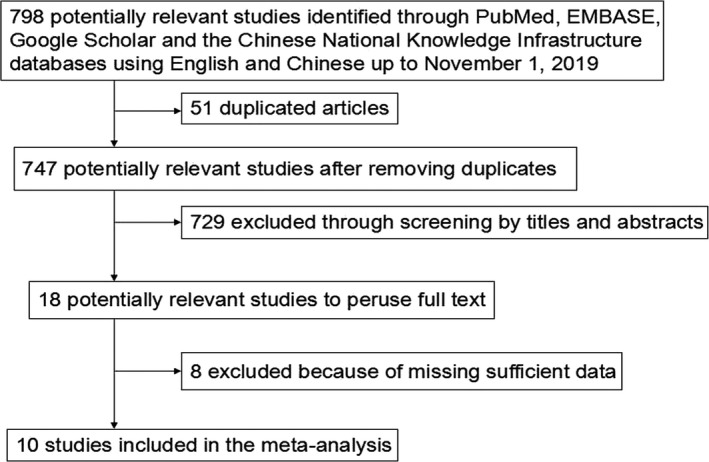
Flow diagram of study selection for meta‐analysis

The literature search identified 798 relevant articles, 51 of which were excluded as duplicated publications. After a preliminary review of titles and abstracts, a total of 747 articles (reviews, case reports, letters, and studies not solely focused on HCC and/or not specifically pertaining to MDK) were excluded for various reasons. The full‐text articles of the 18 remaining publications were obtained, and eight studies were excluded for insufficient data. Finally, 10 publications were selected for our meta‐analysis (Dai et al., 2003; Hodeib, ELshora, Selim, Sabry, & El‐Ashry, 2017; Jia, 2006; Li et al., 2006; Luo et al., 2002; Saad et al., 2013; Shaheen, Abdel‐Mageed, Safwat, & AlBreedy, 2015; Vongsuvanh et al., 2016; Wang, Teng, Wang, Duan, & Pan, 2006; Zhu et al., 2013) (Table 1). The flow diagram of study selection is summarized in Figure 1.

**Table 1:** Main characteristics of the included studies

First author, year	Country	Ethnicity	Method	Patients with HCC/controls	Midkine				Cut‐off	Sensitivity (100%)	Specificity (100%)
					TP	FP	FN	TN			
Luo et al. (2002)	China	Asian	ISHH	33/10	27	0	6	10	NK	81.8	100
Dai et al. (2003)	China	Asian	ISHH	64/26	46	0	16	10	NK	74.2	100
Jia (2006)	China	Asian	ELISA	64/26	64	2	0	24	NK	100	92.3
Wang et al. (2006)	China	Asian	ELISA	46/32	33	5	13	27	3.17 ng/ml	71.7	84.4
Li et al. (2006)	China	Asian	ELISA	104/60	87	8	17	52	70 ng/L	83.7	86.7
Saad et al. (2013)	Egypt	Caucasian	TaqMan	29/45	26	23	3	22	0.302	89.0	64.0
Zhu et al. (2013)	China	Asian	ELISA	252/455	231	94	21	361	0.590 ng/ml	91.7	79.3
Shaheen et al. (2015)	Egypt	Caucasian	ELISA	40/30	37	5	3	25	0.387 ng/ml	92.5	83.3
Vongsuvanh et al. (2016)	Australia	Caucasian	ELISA	86/258	61	98	25	160	0.44 ng/ml	70.9	62.2
Hodeib et al. (2017)	Egypt	Caucasian	ELISA	35/35	34	1	1	34	0.65 ng/ml	98.4	96.2

Abbreviations: FN, false negative; FP, false positive; HCC, hepatocellular carcinoma; NK, not known; TN, true negative; TP, true positive.

### 3.2 Quality assessment of the studies

**Figure 2:**
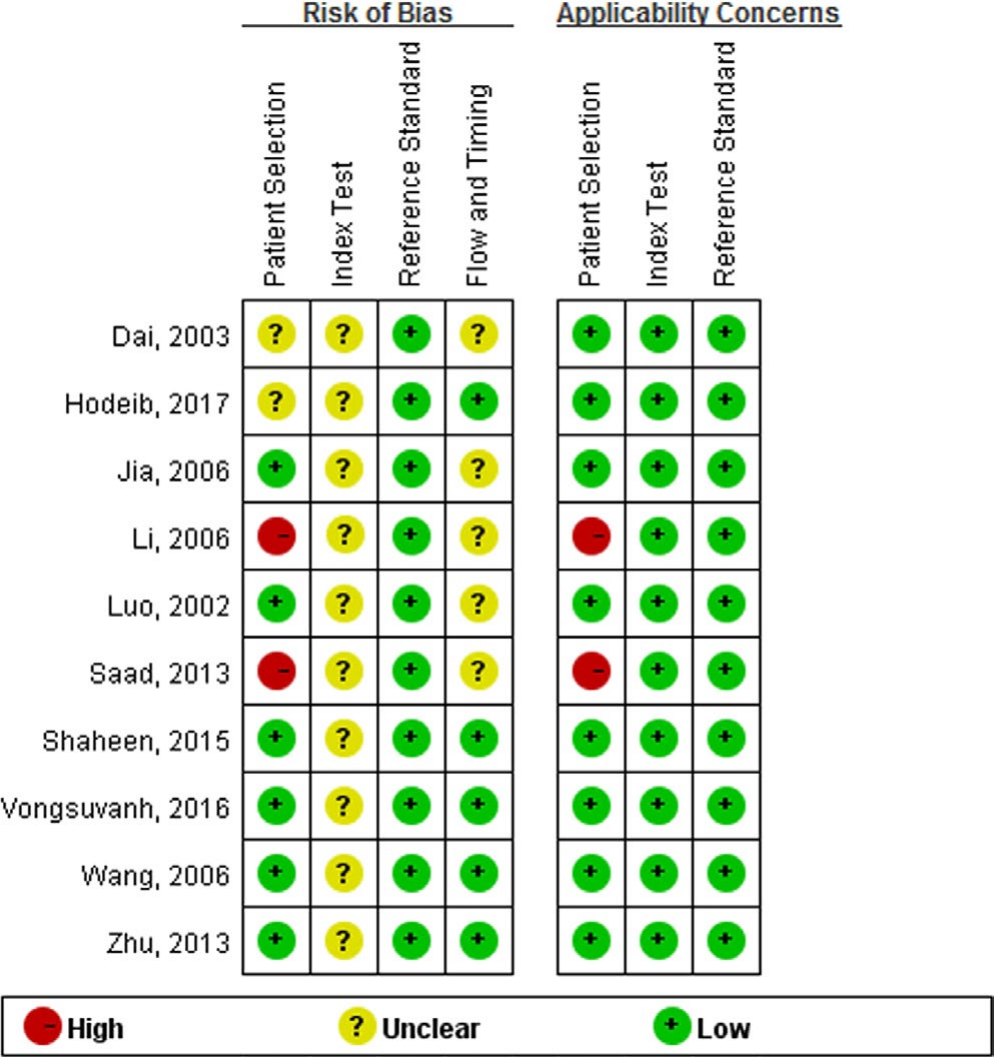
Summary assessment of methodological quality of included studies by Quality Assessment of Studies of Diagnostic Accuracy II

QUADAS‐2 quality assessment of the included studies is shown in Figure 2. All 10 articles were quality assessed. The risk biases of the patient selection domain in two studies (Li et al., 2006; Saad et al., 2013) were labeled as ‘‘high risk’’. None of the studies clearly stated whether the diagnostic thresholds were prespecified, so the risk biases of the index test domain for all studies were labeled as ‘‘unclear’’. Only five (Hodeib et al., 2017; Shaheen et al., 2015; Vongsuvanh et al., 2016; Wang et al., 2006; Zhu et al., 2013) of the studies clearly stated whether all patients received the same reference standard and whether all patients were included in the analysis, thus the risk biases of the rest in the flow and timing domain were also labeled as ‘‘unclear.’’ To investigate the possible source for heterogeneity, we did not exclude the studies that had a high risk of bias or concerns about applicability.

### 3.3 Diagnostic accuracy of MDK

**Figure 3:**
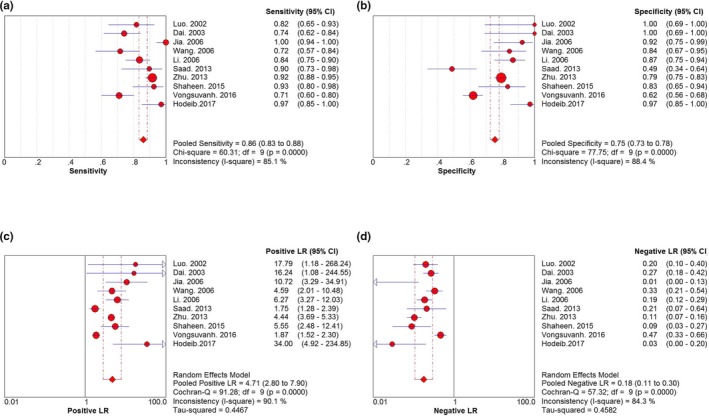
Forest plots of the meta‐analysis of (a) sensitivity, (b) specifcity, (c) PLR, and (d) NLR. PLR, positive likelihood ratio; NLR, negative likelihood ratio

The sensitivity and specificity of each study are shown in Figure 3. The pooled sensitivity and specificity of midkine for the diagnosis of HCC were 86.0% (83.3%–88.4%) and 75.4% (72.6%–78.1%), respectively. The results corresponded to a PLR of 4.70 (2.80–7.90) and an NLR of 0.18 (0.11–0.30) (Figure 3).

**Figure 4:**
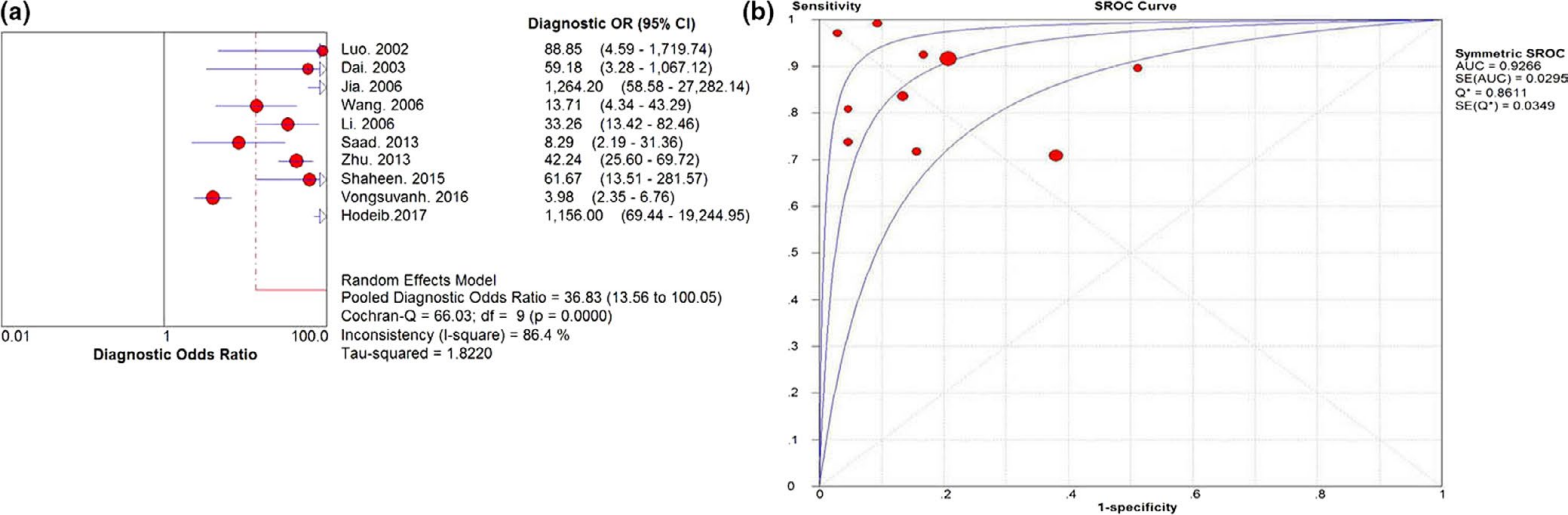
DOR (a) and SROC (b) curve with AUC for midkine. AUC, area under the curve; DOR, diagnostic odds ratio; SROC, summary receiver operator characteristic

The DOR is a single indicator of test accuracy that incorporates sensitivity and specificity into a single index. Unlike sensitivity, specificity PLR, and NLR, this test performance characteristic is not affected by the prevalence of the target disease (Jia, Liu, Gao, Huang, & Du, 2014; Li, Chen, Wang, & Zhang, 2015). The pooled DOR of midkine was 36.827 (95% CI: 13.556–100.05) (Figure 4). The SROC curve of midkine for HCC diagnosis is shown in Figure 4. The area under curve (AUC) was 0.9266 (*SE* = 0.0295) and the Q value for SROC curve was 0.8611 (*SE* = 0.0349).

### 3.4 Threshold effect and heterogeneity

The I‐square of sensitivity, specificity, PLR, NLR, and DOR were 85.1%, 88.4%, 90.1%, 84.3%, and 86.4%, respectively. These results indicated that great heterogeneity existed among eligible studies. To verify whether the heterogeneity could be explained by a threshold effect, the Spearman approach was applied. A value of −0.219 (*p* = .544) indicated the absence of the threshold effect in our meta‐analysis.

With the exception of variances originating from the threshold effect, heterogeneity can be caused by other factors, such as different clinical or sociodemographic characteristics and differences in the study design (Kew, 2010). Meta‐regression analysis was employed to investigate the possible sources of heterogeneity generated by the non‐threshold effect. We initially considered two factors that may contribute to heterogeneity, namely, ethnicity and method. A meta‐regression analysis was performed to confirm whether ethnicity and method were sources of heterogeneity (Table 2). Insignificant heterogeneity was noted among studies in terms of ethnicity (coefficient = −0.357, *p* = .783) and method (coefficient = −2.838, *p* = .173). Therefore, ethnicity and method were probably not the sources of heterogeneity, and others factors might have caused the observed heterogeneity.

**Table 2:** Meta‐regression analysis of the effects of midkine on diagnostic accuracy

Variables	Midkine				
	Coeff.	Std. Err.	*p* value	RDOR	95% CI
Ethnicity	−0.357	1.2430	.7836	0.70	0.03–14.65
Method	−2.838	1.8338	.1728	0.06	0.00–5.21

Abbreviations: CI, confidence interval; RDOR, ratio of diagnostic odds ratio.

## 4 DISCUSSION

Early diagnosis of HCC, which is directly related to therapeutic effects and prognosis, is of vital important. At present, AFP is widely used in clinical practice as the major serum marker for diagnosis of HCC. However, limited by low sensitivity, novel, and reliable markers to complement AFP are urgently needed to improve the diagnostic accuracy for HCC. As one of the novel promising markers (Capurro et al., 2003; Li, Mallory, & Satomura, 2001; Marrero & Lok, 2004; Ozkan et al., 2011; Riener et al., 2009; Yoshida et al., 2002), midkine has been investigated most frequently in recent years (Dai et al., 2003; Hodeib et al., 2017; Jia, 2006; Li et al., 2006; Luo et al., 2002; Saad et al., 2013; Shaheen et al., 2015; Vongsuvanh et al., 2016; Wang et al., 2006; Zhu et al., 2013).

In the present systematic review and meta‐analysis, 10 studies fulfilling the included and excluded criteria which included 1,730 subjects, 753 with HCC and 977 without HCC, were evaluated. Heterogeneity (with the exception of the threshold effect) was found in these studies. The pooled sensitivity and specificity were 86% (95% CI: 83%–88%) and 75% (95% CI: 73%–78%), respectively. The pooled PLR and NLR were 4.71 (95% CI: 2.80–7.90) and 0.18 (95% CI: 0.11–0.30). The pooled DOR and AUC were 36.83 (95% CI: 13.56–100.05) and 0.9266, respectively. All these results indicated that midkin had moderate diagnostic accuracy for HCC.

The PLR in this study was 4.71, indicating that patients with HCC had more than a 4‐fold higher chance of a positive midkine compared to patients without HCC. The NLR was 0.18, which indicated that if the midkine was negative, the probability of these patients developing HCC was approximately 18%. Thus MDK‐negative results may not be used to exclude HCC. DOR converts the strengths of sensitivity and specificity into a single index that represents diagnostic accuracy. DOR is defined as the ratio of the odds of positive test results of participants with a disease to the odds of positive test results of participants without that disease (Duval & Tweedie, 2000). DOR values range from 0 to infinity, with a higher value indicating higher accuracy. In this meta‐analysis, the pooledn DOR of midkine was 36.83, suggesting that midkine showed moderate accuracy in the diagnosis of HCC. The SROC curve and AUC are important for assessing diagnostic data in meta‐analyses. In SROC curve analysis, the emphasis is on a comprehensive evaluation of a diagnostic method and not on simply the method's sensitivity or specificity (Jia et al., 2014; Li et al., 2015; Yang et al., 2015). The AUC is a useful and widely used index of the SROC curve in meta‐analyses and it ranges from 1, which indicates a perfect test that correctly classifies all cases and non‐cases, to 0, which indicates a test that does not perform an accurate diagnosis. The AUC also shows extremely steady performance in heterogeneity tests. In our meta‐analysis, the AUC of midkine were 0.9266, indicating that midkine showed moderate accuracy in the diagnosis of HCC.

Significant heterogeneity was noted in this meta‐analysis, and the value of the Spearman correlation coefficient was −0.219 (*p* = .544), which indicated the lack of heterogeneity caused by the threshold effect. Meta‐regression analyses suggested that ethnicity and testing method may not be the potential sources of heterogeneity among the studies, suggesting that the influencing factors are complex.

To the best of our knowledge, this is the first meta‐analysis concerning the accuracy of midkine in the diagnosis of HCC. At the same time, the work has several limitations that may affect interpretation of the results. First, most of the sample size is limited, so the clinical application of midkine in the diagnosis of HCC still needs long‐term and follow‐up studies for further validation. Second, our exclusion of unpublished data and of papers published in languages other than English and Chinese may have biased our results. Third, the methods used in the study lack uniform standard, which certainly would affect the results. Thus, more large and well‐designed studies are warranted to re‐evaluate the accuracy of midkine in the diagnosis of HCC using methods in uniform standard. Fourth, only two studies (Shaheen et al., 2015; Vongsuvanh et al., 2016) mentioned that all the cases of HCC included were all at early stage (BCLC 0‐A), suggesting that the number of patients with early stage HCC was not specifically mentioned and relatively too small. Therefore, the accuracy of midkine in the diagnosis of HCC at early stage needs to be further investigated.

In conclusion, midkine showed a strong positive correlation with HCC, and the meta‐analysis indicated that midkine had a moderate diagnostic accuracy for HCC. The measurement of midkine may be an optional method in the diagnosis of HCC. More studies with a rigorous design, large sample size, and multiregional cooperation are needed to obtain further evidence on the value of midkine in HCC diagnosis.

## CONFLICTS OF INTEREST

The authors declare no conflicts of interest.

## AUTHOR CONTRIBUTIONS

Designed the study: Le‐Qun Li and Shao‐Liang Zhu. Searched databases and collected full‐text papers: Yu Zhang. Extracted and analyzed the data: Juan Tang. Statistical analyses: Xiao Zhou. Wrote the manuscript: Yu Zhang, Juan Tang, and Xiao Zhou. All authors reviewed the manuscript.
